# Multi-Scale Molecular Photoacoustic Tomography of Gene Expression

**DOI:** 10.1371/journal.pone.0043999

**Published:** 2012-08-27

**Authors:** Xin Cai, Li Li, Arie Krumholz, Zijian Guo, Todd N. Erpelding, Chi Zhang, Yu Zhang, Younan Xia, Lihong V. Wang

**Affiliations:** 1 Department of Biomedical Engineering, Washington University in St. Louis, St. Louis, Missouri, United States of America; 2 Philips Research North America, Briarcliff Manor, New York, United States of America; Shantou University Medical College, China

## Abstract

Photoacoustic tomography (PAT) is a molecular imaging technology. Unlike conventional reporter gene imaging, which is usually based on fluorescence, photoacoustic reporter gene imaging relies only on optical absorption. This work demonstrates several key merits of PAT using *lacZ*, one of the most widely used reporter genes in biology. We show that the expression of *lacZ* can be imaged by PAT as deep as 5.0 cm in biological tissue, with resolutions of ∼1.0 mm and ∼0.4 mm in the lateral and axial directions, respectively. We further demonstrate non-invasive, simultaneous imaging of a *lacZ*-expressing tumor and its surrounding microvasculature *in vivo* by dual-wavelength acoustic-resolution photoacoustic microscopy (AR-PAM), with a lateral resolution of 45 µm and an axial resolution of 15 µm. Finally, using optical-resolution photoacoustic microscopy (OR-PAM), we show intra-cellular localization of *lacZ* expression, with a lateral resolution of a fraction of a micron. These results suggest that PAT is a complementary tool to conventional optical fluorescence imaging of reporter genes for linking biological studies from the microscopic to the macroscopic scales.

## Introduction

A key finding of the Human Genome Project, completed in 2003, was that in our enormous genome of 20,000–25,000 genes, only 2% of the total encodes all of the proteins necessary for building the human body and executing its physiological functions [1]. We are now being challenged to identify the functions of the discovered genes, understand the molecular mechanisms of physiology and pathology, and develop personalized treatment of diseases [2]. These investigations require evaluating the expression patterns of the genes of interest. Traditional methods that measure mRNA *in vitro* have several limitations. First, *in vitro* observation may not correspond to what occurs *in vivo* due to the difficulty of replicating the native microenvironment. Second, they produce only a single data point from each cell culture or sacrificed animal. To get statistically robust data, procedures must be repeated many times, which is labor-intensive, time-consuming, and costly. Third, they are incapable of localizing where genes are expressed in cells. Fourth, due to the various translational and post-translational modifications of the gene products, the level of mRNA for a gene may not quantitatively correlate with the protein production of the same gene [3].

With the promise of overcoming these limitations, molecular imaging is rapidly being developed for interrogating gene expression *in vivo* [4], [5], [6], which allows visualization of the spatiotemporal distribution of gene expression in the native environment. The non-invasive nature of molecular imaging allows for the animals studied to serve as their own controls in a longitudinal study, minimizing the uncertainty caused by inter-sample variability. Thus, molecular imaging greatly reduces animal use, labor, and cost. Moreover, molecular imaging could deeply impact clinical practices by contributing to the early detection and fast staging of diseases, accurate evaluation of treatment outcomes, and rational design of novel therapies.

Two optical imaging tools, bioluminescence imaging (BLI) and fluorescence imaging (FLI), are currently the main techniques used in preclinical research to study gene expression. BLI can detect fM of protein while FLI has a typical sensitivity on the order of nM [5]. Both methods have high throughput rates, however they have limited imaging depth (∼1 cm) [5], lack of depth resolution, and poor lateral resolution. Although µm-order resolution is achievable optically within a thin slide of tissue, deeper structures are poorly resolved *in vivo* due to optical diffusion.

None of the current mainstream molecular imaging modalities can visualize gene expression at both microscopic and macroscopic levels. Previous works have suggested a new approach to imaging gene expression *in vivo* using photoacoustic tomography (PAT) based on optical absorption [7], [8], [9]. In our previous work, a single-scale single−/dual-wavelength photoacoustic system was used to non-invasively and simultaneously image the morphology of a *lacZ*-marked 9 L gliosarcoma and its surrounding microvasculature *in vivo* [7], [8]. Different implementations of PAT allow the spatial resolution to be scaled with the desired imaging depth while maintaining a high depth-to-resolution ratio, which emphasizes the unique multi-scale, high-resolution imaging capability of PAT in this paper [10], [11], [12]. In this work, we demonstrate that PAT can create multi-scale images of gene expression in living biological structures. We show dual-wavelength photoacoustic images with improved image quality and three-dimensional (3D) depiction of the tumor and surrounding vessels. We explore how deep in tissue PAT can detect *lacZ* expression, and also show intra-cellular localization of *lacZ* expression with a sub-micron lateral resolution. The results indicate that PAT can bridge the gap between the microscopic and macroscopic domains in molecular biology studies.

## Materials and Methods

### Chromogenic *lacZ* Reporter Gene System

Although developed earlier, chromogenic reporter genes have been much neglected for molecular imaging, largely due to the lack of tools to visualize their expression inside living organisms. PAT remedies this and, in our opinion, will rejuvenate the development of new *in vivo* chromogenic reporter gene systems to solve current challenges in biological imaging.

**Figure 1:**
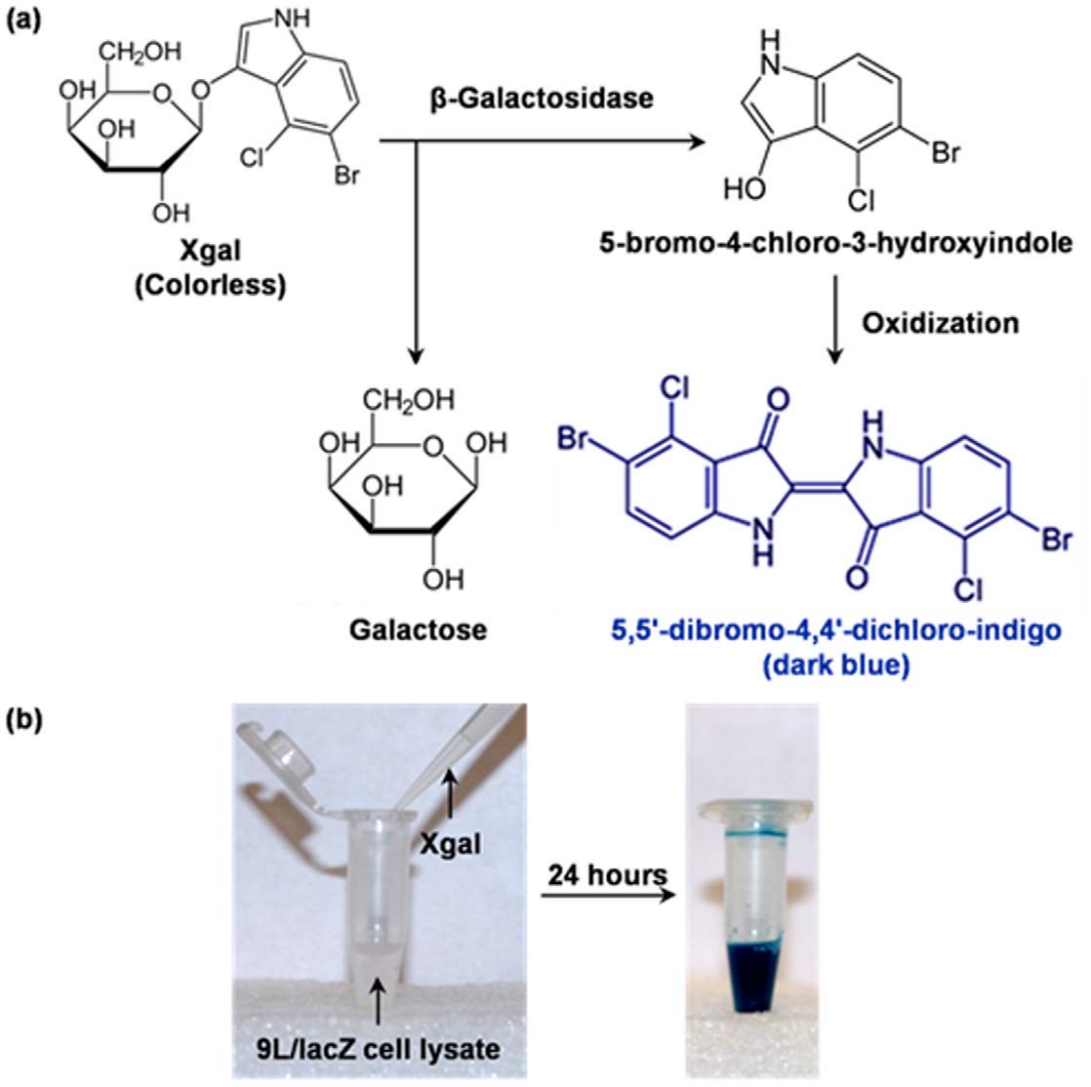
Detecting the expression of the *lacZ* reporter gene using the chromogenic X-gal probe. (a) The hydrolysis of X-Gal catalyzed by β-galactosidase. (b) Photographs showing the chromogenic change after addition of X-gal solution into the native lysate of 9 L/*lacZ* cells.

In this study, we selected the *lac*Z gene, one of the most widely used chromogenic reporter genes, for multiscale PAT. Originating from *E. Coli*, *lacZ* encodes β-galactosidase, a bacterial enzyme responsible for metabolizing lactose into glucose and galactose. The expression of *lacZ* can be studied by several chromogenic assays. Among them, we chose to use 5-bromo-4-chloro-3-indolyl-β-D-galactoside (X-gal) as our *in vivo* molecular probe. X-gal is a colorless analogue of lactose, in which the glucose fragment is replaced by an indole-derivative. β-galactosidase cleaves the glycosidic linkage in X-gal and yields galactose and an optical transparent indoxyl monomer, 5-bromo-4-chloro-3-hydroxyindole. Afterwards, two of the monomers are oxidized to form a stable insoluble blue product, 5,5′-dibromo-4,4′-dichloro-indigo [Figure 1(a)] [13]. Figure 1(b) shows the dramatic chromogenic color change 24 hours after addition of 20 mg/ml X-gal solution to the native cell lysate of transgenic 9 L gliosarcoma cells expressing the *lacZ* reporter gene. There are other colorimetric assays for β-galactosidase activity, such as o-nitrophenyl-β-D-galactoside (ONPG). However, the blue product from X-gal absorbs light strongly between 605 and 665 nm [8] which is located in the so-called “optical diagnostic window”, and allows us to detect the expression of *lacZ* at great depths in tissue.

For *in vivo* imaging, the *lacZ*/X-gal reporter gene system has two noticeable advantages. First, X-gal is an activatable probe. X-gal alone is colorless. Strong optical absorption is generated only after it is cleaved by β-galactosidase. Thus, clearance of the un-cleaved X-gal before imaging is not required. As a result, the activity of β-galactosidase can be measured by PAT against a minimal background. Second, this enzymatic reporter system possesses an intrinsic signal-amplification mechanism. As an enzyme, a single β-galactosidase molecule can cleave multiple X-gal molecules to produce a large number of blue product molecules, allowing us to detect a low expression level of *lacZ*.

### Cell Culture

A wild-type 9 L gliosarcoma cell line was provided by Dr. Michael Welch’s laboratory in the School of Medicine of Washington University in St. Louis (Commercial source: ATCC, Manassas, VA). The 9 L/*lacZ* cell line was created by transfecting the 9 L cell with BAG replication deficient retroviral vector carrying the *E. Coli lacZ* gene. The 9 L/*lacZ* cells were purchased from American Type Culture Center (Manassas, VA). The cells were cultured in Dulbecco’s Modified Eagle Medium (DMEM, Invitrogen, Carlsbad, CA) supplemented with 10% fetal bovine serum (FBS, Invitrogen) and 1 mM sodium pyruvate (Invitrogen).

### Preparation of the Native Cell Lysate

After removal of the culture medium, the 9 L/*lacZ* cells were rinsed with phosphate-buffered saline (PBS, Invitrogen) and then collected into a 15 ml centrifuge tube. The cells were harvested after centrifuging at 4°C for 5 minutes and discarding the supernatant. Then we mixed ice-cold RIPA lysis buffer (Sigma-Aldrich, St. Louis, MO) with the cells and incubated them in ice for 40 minutes. During the incubation, the solution was re-suspended every 10 minutes. Finally, the solution was centrifuged for 20 minutes at 4°C. The supernatant, containing the active natively expressed β-galactosidase, was collected as the lysate of 9 L/*lacZ* cells.

### Animal Preparation for Imaging

This animal study was approved by the Washington University in St. Louis Animal Studies Committee. For tumor inoculation, a 5-µl PBS suspension containing 5 million wild-type 9 L or 9 L/*lacZ* cells was subcutaneously injected into anaesthetized mice (Hsd:Athymic Nude-FoxnlNU, Harlan) using a 0.3-ml syringe with a 29-gauge needle. Tumors were left to grow and monitored daily. Imaging was performed after the progression of the tumor became obvious. Hair was removed before imaging. For detecting the expression of *lacZ*, ∼0.4 mg of X-gal in 20 µl PBS and DMSO (1∶1) was injected near the tumor 1 day before imaging. During imaging, the animals were was maintained under anesthesia by a gas mixture of oxygen and 1% isoflurane flowing at a rate of 1 L/min, and their blood oxygenation and heart rates were closely monitored. All mice recovered naturally after the experiments without photodamage.

### Macroscopic Photoacoustic Imaging [14]

This system is capable of performing both photoacoustic and ultrasonic imaging of the same sample. The photoacoustic signal was excited by 6.5-ns laser pulses generated at a 10-Hz repetition rate and a wavelength of 650 nm by a tunable dye laser (PrecisionScan-P, Sirah) pumped by a Q-switched Nd:YAG laser (PRO-350-10, Newport). Light fluence on the tissue surface was measured to be ∼2 mJ/cm^2^, 10 times lower than the ANSI safety limit (20 mJ/cm^2^). The acoustic signal was detected by a 128-element linear ultrasonic array with a nominal bandwidth of 4–8 MHz (L8-4, Philips Healthcare). The imaging probe was scanned along the elevational direction to obtain a volumetric image. For each 2D frame of the photoacoustic image, measurements were averaged for 100 times, requiring an acquisition time of 10 s. Photoacoustic images were reconstructed using a Fourier-domain beam-forming algorithm [15]. The in-plane resolutions of this system are ∼1.0 mm and ∼0.4 mm in the lateral and axial directions, respectively, at 5 cm deep in biological tissue [16].

### Acoustic-resolution Photoacoustic Microscopy (AR-PAM) [8], [17]

A tunable dye laser (CBR-D, Sirah), pumped by a Nd:YLF laser (INNOSLAB, Edgewave), provided laser pulses at two wavelengths, 584 nm and 635 nm. The pulse width was 7 ns, and the maximum repetition rate was ∼5 kHz. The light focus coaxially overlapped with the focus of a high-frequency ultrasonic transducer (V214-BB-RM, Olympus NDT. Central frequency: 50 MHz). The incident energy density at the tissue surface was controlled to be below 6 mJ/cm^2^. A mechanical stage drove the raster scanning of the imaging probe to obtain a volumetric dataset without averaging. The maximum photoacoustic amplitudes along each axial line were projected onto the skin surface to form a maximum-amplitude projection (MAP) image. The current system was quantified to have a lateral resolution of 45 µm and an axial resolution of 15 µm, and was capable of imaging ∼3 mm deep into the skin [17].

### Optical-resolution Photoacoustic Microscopy (OR-PAM) [18]

For photoacoustic excitation, a compact diode-pumped Nd:YVO_4_ laser (SPOT 100–532, Elforlight, UK. Maximal pulse repetition rate: 50 kHz) generated 1.2-ns pulses at a wavelength of 532 nm. The light was delivered by a single-mode optical fiber and focused into a diffraction-limited spot inside the tissue. The generated photoacoustic signal was detected by an ultrasonic transducer (Central frequency: 40 MHz; NA: 0.5) in transmission mode. The scanning mode was the same as AR-PAM and could also form an MAP image after scanning. The lateral resolution of this set-up was quantified to be 0.40 µm [18].

## Results

### Macroscopic Photoacoustic Imaging

**Figure 2:**
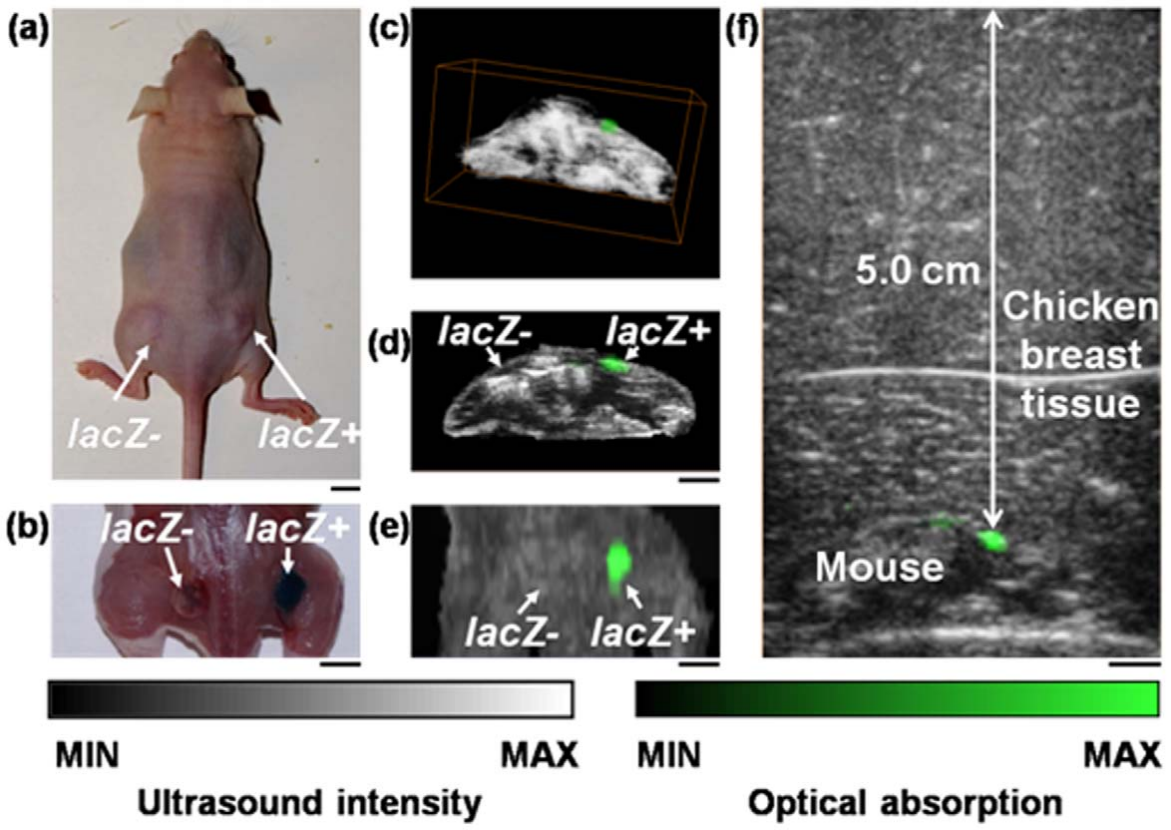
Imaging 9 L tumors with and without the *lacZ* reporter gene by an array-based photoacoustic and ultrasonic imaging system *in vivo*. (a) Photograph of a tumor-bearing mouse before imaging. (b) Post-euthanasia photograph of the *lacZ*- and *lacZ*+ tumors after removing the skin above. (c, d, e) Composite photoacoustic and ultrasonic images shown in (c) three-dimensional visualization, (d) a typical B-scan, and (e) the maximum amplitude projection view on the skin. (f) B-scan image of the *lacZ*-marked tumor at a 5-cm depth in biological tissue, acquired by overlaying chicken breast tissue on top of a mouse. Photoacoustic images are colored green, while ultrasonic images are in gray. The scale bars represent 5 mm.

Figure 2(a) shows a photograph of a tumor-bearing nude mouse before imaging. 9 L tumors with and without *lacZ* transgene were grown in its right and left flanks, respectively. Both tumors were injected with the same amount of X-gal before imaging. Once the mouse was sacrificed after imaging, we removed the skin on top of the tumors, and found only the *lacZ* + tumor was stained blue, which further confirms the specificity of the detection of the *lacZ* expression by the chromogenic X-gal probe [Figure 2(b)]. The tumor-bearing region of the mouse was imaged *in vivo* with both photoacoustic and ultrasonic imaging. Figures 2(c–e) show the composite dual-modality images in volumetric visualization, a cross-sectional view, and projection view, respectively. Therein, photoacoustic images are colored green, while ultrasonic images are colored in gray scale. Figure 2(c) shows we could localize the *lacZ* + tumor in three dimensions. Also, we observed that although the tumor showed as a hypoechoic area in ultrasonic images, the lack of ultrasonic scattering was not a specific indicator for tumor [Figure 2(d)]. In contrast, using the *lacZ*/X-gal reporter strategy, PAT could detect the *lacZ*-marked tumor with a high specificity [Figure 2(e)]. Moreover, by overlaying chicken breast tissue on top of the tumor, we found the expression of *lacZ* remained visible at a depth of 5.0 cm in biological tissue, with a contrast of 3.04.

### Acoustic-resolution Photoacoustic Microscopy

**Figure 3:**
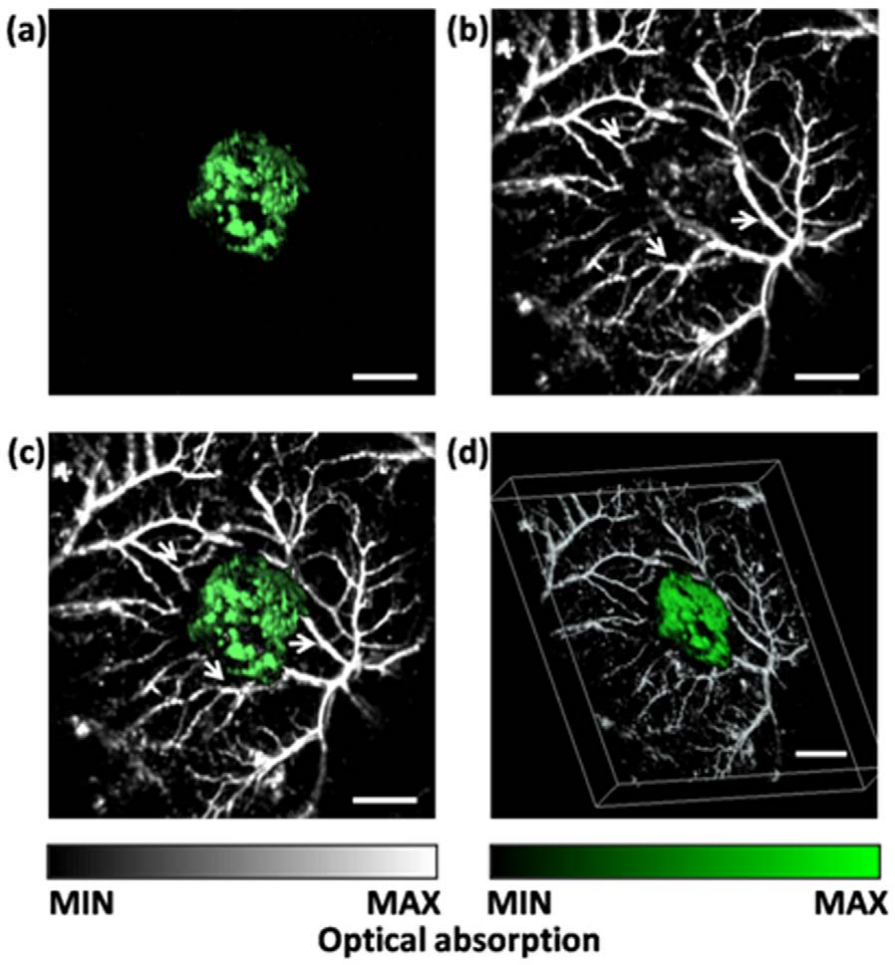
Simultaneously imaging a *lacZ*-marked tumor and its associated microvasculature by dual-wavelength AR-PAM *in vivo*. (a) MAP mage acquired at 635 nm showing tumor morphology. (b) MAP image acquired at 584 nm showing microvasculature. (c) Composite image showing the spatial relations between tumor and blood vessels. (d) 3D depiction of composite PA image showing the tumor and blood vessels (Video S1, MPEG, 5.75 MB). Green: tumor. Arrows indicate feeding vessels of the tumor. The scale bars represent 2 mm.

Blood vessels and the blue product have well-separated absorption peaks, and can be separately visualized by PAT at different optical wavelengths (Figure 3). Two wavelengths (584 nm and 635 nm) were selected to maximize the difference between the optical absorption of hemoglobin and the blue product within the efficient emission band of the DCM laser dye. While the 635-nm wavelength was selected to map the *lacZ*-marked tumor [Figure 3(a)], the 584-nm wavelength was used to visualize the microvasculature [Figure 3(b)]. The photoacoustic signal in Figure 3(b) represents the relative value of total hemoglobin concentration. A combined image [Figure 3(c) and 3(d)] shows the spatial relation between the tumor and the surrounding microvasculature.

### Optical-resolution Photoacoustic Microscopy

**Figure 4:**
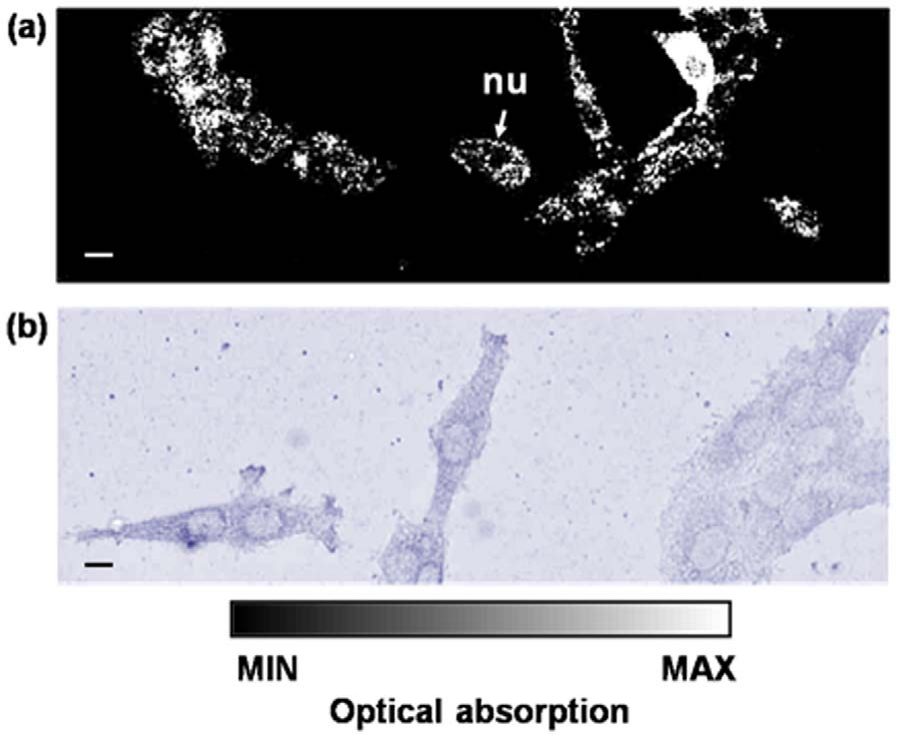
Imaging 9 L/lacZ cells stained with X-gal (a) MAP image by OR-PAM. (b) Micrograph. nu: cell nucleus. The scale bars represent 10 µm.

Figure 4(a) shows photoacoustic images of fixed 9 L/*lacZ* cells grown on a cover glass after staining in 1 mg/ml X-gal solution at 37°C for 12 hours. Figure 4(b) shows a micrograph of fixed 9 L/*lacZ* cells using a Nanozoomer 2.0-HT slide scanner (Hamamatsu, Hamamatsu City, Japan). We observed that 9 L/*lacZ* cells were between 20 and 40 µm in diameter. Inside the cells, strong absorbers were scattered around low-absorbing centers that are cell nuclei (nu, indicated by arrow). This finding implies that β-galactosidase, the final product of *lacZ* expression, exists mostly in the cytoplasm.

## Discussion

Using the macroscopic photoacoustic system, we imaged the expression of *lacZ* at a depth of 5.0 cm in biological tissue. This demonstrated the advantage of PAT over other optical molecular imaging techniques, such as BLI and FLI, in penetration depth. In addition, all images were obtained by averaging over 100 measurements, but using a low optical energy at 1/10 of the ANSI exposure limit. Assuming noise was uncorrelated between measurements, we could potentially obtain images with the same quality at a speed of 10 frames/second while using the maximum laser energy allowed by the ANSI standard. The imaging speed can be further improved by adopting a high-repetition rate laser.

We further demonstrated simultaneous imaging of a *lacZ*-marked tumor and its surrounding microvasculature by dual-wavelength AR-PAM. Remodeling of the microcirculation plays an important role in oncology. Although photoacoustic computed tomography is able to detect the *lacZ*-marked tumor at great depth, it lacks the resolving power to visualize the microcirculation system associated with the tumor. AR-PAM uses a high-frequency focused transducer, and can achieve sufficient resolving power to visualize the microvasculature at the cost of imaging depth.

It is noted that the reaction product of X-gal is almost insoluble in water and is thus difficult to be cleared by the body. However, by using other galactopyranosides (e.g., chlorophenolred-β-D-galactopyranoside (CPRG) and ortho-Nitrophenyl-β-galactoside (ONPG)) whose cleavage products by β-galactosidase are water-soluble, chronic monitoring of gene expression in a single sample at multiple time points can be achieved.

We showed the sub-cellular localization of the *lacZ* expression using OR-PAM. By confining photoacoustic excitation by tight optical focusing, OR-PAM has been proven to obtain a lateral resolution of a fraction of a micron [18], and could potentially play an important role in studying molecular biology in cultured cells.

Currently, each of the imaging systems is independent, which means one PAT system achieves only one specific resolution. Thus, a single PAT system with switchable resolutions and penetration depths is of great interest. To integrate a macroscopic photoacoustic imaging system with AR-PAM, we simply need to replace the 50-MHz transducer in AR-PAM with a lower frequency transducer and adjust the laser exposure accordingly. Previous work has demonstrated that an AR-PAM with a 5-MHz transducer provides penetration up to 38 mm in chicken breast tissue. At the 19-mm depth, the axial resolution is 144 µm and the lateral resolution is 560 µm [19]. To integrate AR-PAM with OR-PAM, the laser output can be split into two beams, and a fiber bundle can be used to switch illuminations for AR-PAM and OR-PAM. A single core of the fiber bundle can deliver light for OR-PAM with high lateral resolution, while all cores of the fiber bundle can illuminate a larger area for AR-PAM. An integrated AR-PAM and OR-PAM system is under construction by our group.

In summary, we demonstrated several key merits of PAT as a promising candidate for molecular imaging. We proved that the expression of *lacZ* can be detected by PAT as deep as 5.0 cm in biological tissue. In addition, we showed that PAT could follow the gene expression at scalable depths and resolutions. We expect that PAT could become an important tool linking biological studies at the microscopic and macroscopic levels. With the future development of new chromogenic reporter gene systems, photoacoustic reporter gene imaging can impact both laboratory research and clinical practice.

## Supporting Information

Video S1 **3D depiction of composite AR-PAM image acquired at 635 nm and 584 nm showing the tumor and blood vessels.** Green: tumor. Red: microvasculature.
(MPG)

## References

[1] International Human Genome Sequencing Consortium (2004) Finishing the euchromatic sequence of the human genome. Nature 431: 931–945. 15496913 10.1038/nature03001

[2] Collins FS, Green ED, Guttmacher AE, Guyer MS (2003) A vision for the future of genomics research. Nature 422: 835–847. 12695777 10.1038/nature01626

[3] Taniguchi Y, Choi PJ, Li GW, Chen HY, Babu M, et al (2010) Quantifying E-coli Proteome and Transcriptome with Single-Molecule Sensitivity in Single Cells. Science 329: 533–538. 20671182 10.1126/science.1188308 PMC2922915

[4] Weissleder R, Mahmood U (2001) Molecular imaging. Radiology 219: 316–333. 11323453 10.1148/radiology.219.2.r01ma19316

[5] Massoud TF, Gambhir SS (2003) Molecular imaging in living subjects: seeing fundamental biological processes in a new light. Genes & Development 17: 545–580. 12629038 10.1101/gad.1047403

[6] Herschman HR (2003) Molecular imaging: Looking at problems, seeing solutions. Science 302: 605–608. 14576425 10.1126/science.1090585

[7] Li L, Zemp RJ, Lungu G, Stoica G, Wang LV (2007) Photoacoustic imaging of lacZ gene expression *in vivo* . Journal of Biomedical Optics 12: 020504. 17477703 10.1117/1.2717531

[8] Li L, Zhang HF, Zemp RJ, Wang LV (2008) Simultaneous imaging of a lacZ-marked tumor and microvasculature morphology *in vivo* by dual-wavelength photoacoustic microscopy. Journal of Innovative Optical Health Sciences 1: 207–215. 19946613 10.1142/S1793545808000212 PMC2782593

[9] Filonov GS, Krumholz A, Xia J, Yao J, Wang LV, Verkhusha VV (2012) Deep-tissue photoacoustic tomography of a genetically encoded near-infrared fluorescent probe. Angewandte Chemie International Edition 51(6), 1448–1451. 10.1002/anie.201107026 PMC3293502 22213541

[10] Wang LV (2009) Multiscale photoacoustic microscopy and computed tomography. Nature Photonics 3: 503–509. 20161535 10.1038/nphoton.2009.157 PMC2802217

[11] Beard P, Arridge S, Cox B, Laufer J (2009) Quantitative Photoacoustic Imaging. Photoacoustic Imaging and Spectroscopy: CRC Press. 121–143.

[12] Ntziachristos V, Razansky D (2010) Molecular Imaging by Means of Multispectral Optoacoustic Tomography (MSOT). Chemical Reviews 110: 2783–2794. 20387910 10.1021/cr9002566

[13] Cepko C, Ryder E, Fekete DM, Bruhn S (1998) Detection of beta-galactosidase and alkaline phosphatase activities in tissue. In: Spector DL, Goldman RD, Leinwand LA, editors. Cells: A Laboratory Manual, Volume 3: Subcellular Location of Genes and Their Products. Cold Spring Harbor Cold Spring Harbor Laboratory Press.

[14] Kim C, Erpelding TN, Jankovic L, Pashley MD, Wang LV (2010) Deeply penetrating *in vivo* photoacoustic imaging using a clinical ultrasound array system. Biomedical Optics Express 1: 278–284. 21258465 10.1364/BOE.1.000278 PMC3005157

[15] Köstli KP, Frenz M, Bebie H, Weber HP (2001) Temporal backward projection of optoacoustic pressure transients using Fourier transform methods. Physics in Medicine and Biology 46: 1863. 11474930 10.1088/0031-9155/46/7/309

[16] Kim C, Erpelding TN, Jankovic L, Wang LV (2011) Performance benchmarks of an array-based hand-held photoacoustic probe adapted from a clinical ultrasound system for non-invasive sentinel lymph node imaging. Philosophical Transactions of the Royal Society A: Mathematical, Physical and Engineering Sciences 369: 4644–4650. 10.1098/rsta.2010.0353 PMC3263783 22006911

[17] Zhang HF, Maslov K, Stoica G, Wang LV (2006) Functional photoacoustic microscopy for high-resolution and noninvasive *in vivo* imaging. Nature Biotechnology 24: 848–851. 10.1038/nbt1220 16823374

[18] Laufer JG, Cleary JO, Zhang EZ, Lythgoe MF, Beard PC (2010) Photoacoustic imaging of vascular networks in transgenic mice. Proceedings of SPIE 7564: 75641A.

[19] Song K, Wang LV (2007) Deep reflection-mode photoacoustic imaging of biological tissue. Journal of Biomedical Optics 12: 060503. 18163798 10.1117/1.2818045

